# Impact of SNPs/Haplotypes of *IL10* and *IFNG* on the Development of Diffuse Large B-Cell Lymphoma

**DOI:** 10.1155/2019/2137538

**Published:** 2019-11-26

**Authors:** Amanda Vansan Marangon, Cristiane Maria Colli, Daniela Maira Cardozo, Jeane Eliete Laguila Visentainer, Ana Maria Sell, Fernando Guimaraes, Silvia Barbosa Dutra Marques, Sophia Rocha Lieber, Francisco José Penteado Aranha, Roberto Zulli, Victor Hugo de Souza, Carmino Antonio de Souza

**Affiliations:** ^1^Faculdade de Ciências Médicas, Universidade Estadual de Campinas (UNICAMP), Brazil; ^2^Department of Basic Health Sciences, State University of Maringá (UEM), Av. Colombo, 5790, Maringá, Paraná, Brazil; ^3^Postgraduate Program on Bioscience and Physiopathology, UEM, Brazil; ^4^Hospital da Mulher Prof. Dr. José Aristodemo Pinott, Universidade Estadual de Campinas (UNICAMP), Brazil

## Abstract

The purpose of this study was to assess the influence of single-nucleotide polymorphisms (SNPs) on cytokine genes in the development of diffuse large B-cell lymphoma (DLBCL). One hundred and twelve patients and 221 controls were investigated. Among them, 97 patients treated with R-CHOP were subdivided into two groups: (i) complete remission of the disease and (ii) patients who progressed to death, relapsed, or had disease progression. The SNPs investigated by PCR-SSP were *TNF* -308G>A (rs1800629), *IFNG* +874A>T (rs2430561), *IL6* -174G>C (rs1800795), *IL10* -1082A>G (rs1800896), *IL10* -819C>T (rs1800871), *IL10* -592C>A (rs1800872), and *TGFB1* codon10T>C (rs1982073) and codon25G>C (rs1800471). In general, the genotypes that have been associated in the literature with lower production or intermediate production of IL-10 and higher production of IFN-*γ* were associated with the protection of the development of the disease, possibly favoring the Th1 immune response and diminishing the capacity of cell proliferation. However, patients receiving R-CHOP treatment presented unfavorable prognoses in the presence of genotypes related to the intermediate production of IL-10 and high production of TGF-*β*1, indicating that cytokines may be related to the response to treatment and action mechanisms of Rituximab.

## 1. Introduction

Diffuse large B-cell lymphoma (DLBCL) is considered a rare disease, affecting 1-5/10,000 individuals [1], and it is the most common type of non-Hodgkin's lymphoma (NHL). Many cases of DLBCL do not have a definite etiology, but the disease is frequently reported in individuals with autoimmune diseases and acquired immune deficiency or in immunosuppressed patients [2], evidencing the importance of the immune response in this type of lymphoma.

Studies have suggested that immunosuppression and inhibition of Th1 responses are related to the risk of developing lymphomas [3, 4], and several single-nucleotide polymorphisms (SNPs) in cytokine genes have been associated with a higher or lower risk of developing B-cell lymphomas [3, 5–7].

Secretion of certain cytokines from tumor cells, particularly interleukin- (IL-) 6, IL-10, and TNF-*α*, renders them resistant to the cytotoxic effects of chemotherapeutic drugs. The inclusion of Rituximab in the treatment composed of Cyclophosphamide, Doxorubicin, Vincristine, and Prednisone (R-CHOP) greatly improved the chemotherapy response and survival of the DLBCL patients. However, not all patients respond in the same way to the treatment, and the action mechanisms of Rituximab are not fully understood [8–10].

Some authors have shown that polymorphisms in cytokine genes may influence the development of DLBCL, while other researchers have been struggling to find biomarkers that can serve as prognosis and predictors, not only to identify patients who will undergo a particularly aggressive course but also to select with accuracy the most beneficial therapeutic treatment for each patient [8–11].

In this context, the purpose of the present study was to evaluate the influence of polymorphisms on cytokine genes in the development of DLBCL and in the prognosis of patients treated with R-CHOP.

## 2. Materials and Methods

### 2.1. Ethical Aspects

The research was approved by the Ethics in Research Committee involving human beings from the State University of Campinas (UNICAMP No. 1117/2009) attending the Helsinki Declaration of 1975, as revised in 2008. All participants signed a free and informed consent form.

### 2.2. Characterization of Patients and Controls

From April 2009 to June 2013, 112 patients with DLBCL were selected from Campinas and region (22°54′S and 47°03′W) and treated at the State University of Campinas in the Hematology and Hemotherapy Center (CHH-UNICAMP). Clinical and epidemiological data, including age, gender, ECOG (Eastern Cooperative Oncology Group), IPI (International Progression Index), Ann Arbor staging, type of treatment, nodal involvement, LDH (lactate dehydrogenase) levels, bone marrow involvement, and subtype lymphomas, were obtained from each patient's medical records.

The diagnosis of large B-cell non-Hodgkin lymphoma was performed through histopathological examination of the affected lymph node or biopsy of the tumor mass, and the classification of the lymphoma subtype was performed through an immunohistochemical evaluation, as classified by the World Health Organization [12]. The initial staging of patients with lymphoma was performed based on the clinical history and physical examination, as well as on the results obtained from the imaging exams: computed tomography of the chest and abdomen or chest X-ray and abdominal ultrasound and bone marrow biopsy. Patients were monitored during the treatment and posttreatment period with regularly scheduled clinical and radiographic exams. Of the total number of patients, 101 were treated with the R-CHOP treatment (Rituximab, Cyclophosphamide, Doxorubicin, Vincristine, and Prednisone of 4 to 8 cycles, depending on lymphoma staging). Patients were divided into groups according to their response to treatment: Those with normalization of physical and radiological findings four weeks after the last cycle of chemotherapy were considered as patients in complete remission. Those with a reduction of 50% of the initial tumor mass were defined as patients in partial remission, and those who did not present the above criteria were considered nonresponders.

The control group consisted of 221 voluntary donors of blood and bone marrow registered at CHH-UNICAMP from the same region as patients with DLBCL. Pairing was done according to gender.

### 2.3. DNA Extraction and Cytokine Genotyping

Blood samples from each individual were collected in EDTA tubes, and the respective DNAs were extracted using the DNA blood mini kit (QIAamp, Qiagen, Mississauga, Canada), following the manufacturer's recommendations.

Eight SNPs were investigated by Polymerase Chain Reaction-Single Specific Primer (PCR-SSP): *TNF -308G>A* (*rs1800629*), *IFNG +874A>T* (*rs2430561*), *IL6 -174G>C* (*rs1800795*), *IL10 -1082A>G* (*rs1800896*), *IL10 -819C>T* (*rs1800871*), *IL10 -592C>A* (*rs1800872*) and *TGFB1 codon10T>C* (*rs1982073*) and *codon25G>C* (*rs1800471*), using the commercial cytokine genotyping kit (One Lambda, Canoga Park, CA, USA), following the manufacturer's recommendations. The integrity of the reaction was evaluated by a pair of primers as the internal control. Standard forms supplied by the manufacturer were used for the interpretation.

### 2.4. Statistical Analyses

The SNPStats web tool, available at https://www.snpstats.net/start.htm, was used to perform statistical analyses [13]. Genotype and haplotype frequencies were calculated in patient and control samples, and the Hardy-Weinberg equilibrium was evaluated from the genotype distribution analysis. The haplotype frequencies were estimated using the implementation of the expectation-maximization (EM) algorithm coded into the haplo.stats package [13] and were performed for the *IL10* and *TGFB1* genes. A logistic regression test, adjusted for age and gender, was used to verify the association of SNPs in cytokine genes and the development of DLBCL, as well as to evaluate the association of these SNPs in the progression of the disease in individuals treated with R-CHOP. Association tests were performed for codominant, dominant, recessive, overdominant, and log-additive genetic inheritance models. The best inheritance model was chosen according to the lowest Akaike information criterion (AIC). To assess the association of SNPs in the genes studied in the progression of DLBCL, patients treated with R-CHOP were divided into two groups: one group composed of patients who had complete disease remission (control) and the other group with patients who died or had recurrence or progression of the disease. It was also analyzed whether polymorphisms in the cytokine genes influenced in clinical staging (advanced stages of disease versus nonadvanced stages), LDH (normal versus augmented), and IPI (low and intermediate-low versus intermediate-high and high). Contingency tables were constructed, and the chi-squared test was performed to analyze the combined effect between SNPs in different cytokines, with Yates correction when necessary. The odds ratio (OR) was calculated considering a 95% confidence interval, and results were considered significant with *P* < 0.05. The Bonferroni adjustment for multiple testing was not applied because all variants analyzed have been associated with DLBCL in any other populations.

The influence of cytokine genes and the *IL10* ACC haplotype were also investigated on the clinical evolution of DLBCL based on the overall survival and progression-free survival analyses with the Kaplan-Meier estimator. Cox univariate models were used to determine the statistical significance (*P* values of the methods: “likelihood,” “Wald,” and “log-rank”) and the cytokine gene relative risks (RR) with confidence intervals (CI) of 95%, besides other variables as age, gender, LDH, disease stages, IPI, and response to treatment. Factors associated with the risk of progression and death with 20% significance were examined using Cox multivariate analysis. Analyses were conducted with statistical package “R” version 4.0.2. for Windows program [14].

## 3. Results

### 3.1. Characteristics of Patients and Controls

The characteristics of patients and controls are described in Table 1. The mean age of the patients and controls was 60.4 years and 50.6 years, respectively. The majority of the patients were more than 60 years of age, while the majority of the controls were younger than 60 years old (*P* ≤ 0.05).

Of the 101 patients treated with the R-CHOP, 72 (71.2%) presented complete remission, 17 (16.8%) died, four (4.0%) relapsed, four (4.0%) presented disease progression, and four (4.0%) patients were lost to follow-up (Table 1).

Patients were followed up until October 2013. The median time for overall survival was 17 months (1-54 months), and for progression-free survival, it was 16.9 months (1-54 months).

### 3.2. Genotype and Haplotype Frequency Distributions in Patients and Controls

The distribution of observed and expected genotype frequencies for all genes analyzed was in Hardy-Weinberg equilibrium (*P* > 0.05).

The distribution of the genotype and haplotype frequencies of the studied cytokine genes in patients with DLBCL and controls is showed in Table 2. Significant differences were not observed for most of analyzed SNPs between DLBCL patients and controls. However, for *IL10* -819 and *IL10* -592, higher genotype frequencies of the variants were observed in patients when compared to controls. The choice inheritance model was the dominant where a single copy of the mutant allele is sufficient to modify the risk. Through this model, the genotypes C/T or T/T and C/A or A/A of the *IL10 -819* and *IL10* -592, respectively, are indicated to be a risk factor for the development of DLBCL in relation to the C/C genotypes of both SNPs (OR: 2.23, *P* = 0.013). On the other hand, the *IFNG* +874 T/T or A/T genotype frequencies were less frequent in patients than controls (OR: 0.35, *P* < 0.001) and, also, in a dominant inheritance model. The statistical power of the test was on average 88%, ranging from 75 to 97%.

The presence of *IL10* polymorphisms (-819C>T and -592C>A) indicated risk for the development of DLBCL, and the presence of *IFNG* polymorphism (+874A>T) indicated protection. The individuals that presented three genotypes (C/T or T/T and C/A or A/A of the *IL10 -819* and *IL10 -592*, respectively, and the A/A of the *IFNG +874*) that could favor the development of the disease were grouped and compared with the individuals that did not have these three genotypes together. For this combined analysis, these two *IL10* SNPs were being counted as a single one, since they are in strong linkage disequilibrium (*D*′ = 0, 99). It was observed that the risk of developing DLBCL in individuals who had all the three genotypes was higher (OR = 3.98, CI: 2.29-6.14, *P* ≤ 0.01) than when assessing the individual risk of each SNP.

In the R-CHOP-treated patients, considering the prognosis of the disease, the *TGFB1* codon10 T/C genotype frequency was higher in patients who died or had recurrence or progression of the disease compared to patients with complete remission of disease (OR: 2.39, *P* = 0.03, overdominant inheritance model). In the overdominant inheritance model, heterozygous alleles are compared to a pool of both homozygous alleles. The other SNPs studied did not influence the prognosis of the treated patients (Table 3).

In addition to these results, the distribution of the haplotype frequencies of *IL10* and *TGFB1* was analyzed considering the development of DLBCL and the prognosis of the disease in patients treated with R-CHOP, and the results are shown in Tables 2 and 3, respectively. According to *TGFB1*, there were no differences in the haplotype frequency distributions when the development of DLBCL or prognosis of the disease were considered. However, the haplotype *IL10* -1082, -819, and -592 ACC was more frequent in controls compared to DLBCL patients (OR: 0.55, *P* = 0.03; compared to the ATA haplotype), and it was less frequent in patients treated with R-CHOP with complete remission of disease compared to patients who died or had recurrence or progression of the disease to progress to death (OR: 2.83, *P* = 0.03; compared to the ATA haplotype). The SNPs investigated did not influence the clinical staging of patients considering LDH concentration or IPI.

### 3.3. Analysis of Overall Survival and Progression-Free Survival

**Figure 1:**
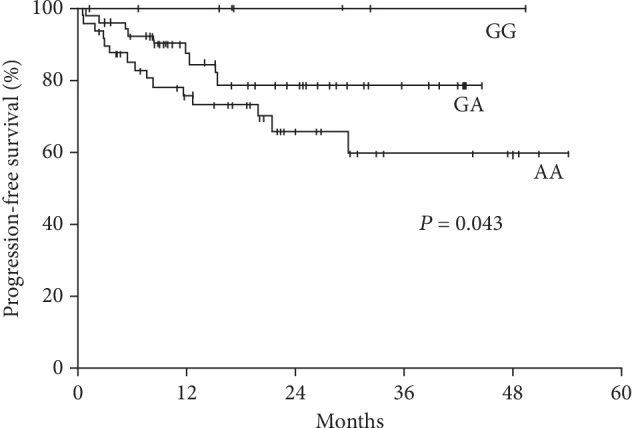
These Kaplan-Meier curves compare progression-free survival in patients with diffuse large B-cell lymphoma according to *IL10* -1082 genotypes A/A, G/A, and G/G.

For the *IL10* -1082 polymorphism, patients with the G/G genotype presented greater progression-free survival than the G/A and A/A genotypes (Figure 1). The other polymorphisms, as well as the presence or absence of the haplotype ACC of *IL10*, had no impact on the clinical evolution of DLBCL based on global and progression-free survival, as well as on the therapeutic response in the univariate and multivariate Cox analyses.

## 4. Discussion

In the present study, we analyzed the possible associations of polymorphisms in cytokine gene representative of Th1 and Th2 immune responses, including *TNF* -308, *IFNG* +874, *IL6 -*174, *IL10* -1082, *IL10* -819, *IL10* -592, and *TGFB1* codon10 and *TGFB1* codon25, with the development of DLBCL and progression of the disease in patients treated with R-CHOP. We found that the C/T or T/T and C/A or A/A genotypes of *IL10 -819* and *IL10* -592, respectively, increased 1.23 times the risk of developing DLBCL in relation to the patients that had the C/C genotypes in the both SNPs. On the other hand, the T/T or T/A genotypes of *IFNG* +874 could be a protective factor in the development of the disease in relation to the A/A genotype. We also observed the association of the ACC haplotype (*IL10* -1082, -819, and -592) of *IL10* as a protective factor for the development of the disease and a risk factor for patients treated with R-CHOP to evolve to death or have relapse or disease progression. With regard to patients treated with R-CHOP, the presence of the *TGFB1* codon10T/C genotype increased 1.39 times the chance of disease progression, recurrence, or death, compared to patients with T/T or C/C genotypes. The genotype associations between cases and controls were strong, since generally a statistical power of 80% ensures a high probability of observing a certain effect. However, the statistical power observed in the associations involving only treated patients was considered low, probably because the number of the subjects in each groups was small and more studies should be performed with more patients.

Polymorphisms in promoter and regulatory regions of cytokine genes have been related to the production and expression of various cytokines [5, 15–18]. In addition, some polymorphisms have been associated with the development or progression of DLBCL [5, 7, 10].

Interleukin-10 is a cytokine with a strong immunosuppressive effect, because it inhibits the proinflammatory action of Th1 lymphocytes [19] and may favor tumor growth *in vitro* by stimulating cell proliferation and inhibiting apoptosis [18]. The role of polymorphisms in the *IL10* gene and its influence on cytokine levels are controversial. The absence of the A-allele in the position -1082 was associated, *in vitro*, with elevated levels of the cytokine independent of polymorphisms at other positions of the gene [15]. However, in another study with DLBCL patients, the frequencies of the T (*IL10* -819) and A (*IL10* -592) alleles were shown to be lower in patients with high levels of IL-10 [4]. In our study, the haplotype ACC (*IL10* -1082, -819, and -592) was a protective factor for the development of the disease but was associated with worse prognosis in patients treated with the R-CHOP regimen when compared to the ATA haplotype. Liu et al. [10] found lower progression-free survival in patients with DLBCL treated with R-CHOP who had this same ACC haplotype when compared to patients who did not. Although in our study the haplotype ACC of *IL10* did not influence the overall or progression-free survival, we observed an association of the genotype G/G (*IL10* -1082) with greater progression-free survival. These results are similar to those observed by Lech-Maranda et al. [5], who found an association between the genotypes G/G and G/A, in this same position, with better overall survival and better complete remission rate in patients with DLBCL. In this study, we also observed that the C/C genotypes of *IL10 -819* and *IL10* -592 were more frequent in controls compared to DLBCL patients, indicating that it was a protective factor. These results are consistent with those observed in relation to the ACC haplotype, which was a protective factor for the development of the disease in relation to the ATA haplotype. This perhaps indicates that polymorphisms in the *IL10 -819* and *IL10* -592 regions are likely to be more important factors in the development of DLBCL.

Interferon-*γ* is considered one of the main endogenous immunoregulators, acting mainly as a proinflammatory cytokine [20]. In the present study, it was demonstrated that the *IFNG* +874 polymorphism seems to have an impact on the development of DLBCL, since the T/T and T/A genotypes were protective factors in relation to the A/A genotype which is considered a low producer of this cytokine [21, 22]. This result is similar to another study in which the A/A genotype was related to the increased risk of developing posttransplant lymphoproliferative disorders [23]. The risk of developing DLBCL was even higher when comparing individuals with three risk genotypes (C/T or T/T and C/A or A/A of the *IL10 -819* and *IL10 -592*, respectively, and the A/A of the *IFNG +874*) to those with only two, one or none of these genotypes. This is probably related to lower proinflammatory cytokine production levels (assigned to A/A of the *IFNG +874*) and IL-10 (anti-inflammatory) production levels, causing a summing effect, increasing the risk of developing the disease. However, further studies need to be done to better understand the association between genotypes and level production and expression of these cytokines.

The T/C genotype of *TGFB1* codon10, considered a high cytokine producer [24], was a risk factor for patients treated with R-CHOP to evolve into a worse prognosis. TGF-*β*1 is a multifunctional anti-inflammatory cytokine that participates in different physiological situations, including cell cycle control, hematopoiesis control, cell differentiation, angiogenesis, induction of apoptosis, and cell matrix formation [25]. In the NHL, the expression of this cytokine is associated with more aggressive lymphomas [9] and, the high-producing genotype of this cytokine was associated with unfavorable prognoses [24].

As our control group consisted of voluntary blood and bone marrow donors with maximum age of 60 years, it was not possible to match the patients and controls by age, considering that DLBCL mainly affects individuals after the age of 60. We acknowledge that this may indicate a flaw in our study, and in order to decrease the bias, we did the logistic regression test adjusted for age and gender.

Generally speaking, genotypes associated with lower production or intermediate production of IL-10 and increased production of IFN-*γ* were associated with the protection for the development of the DLBCL, possibly favoring the Th1 immune response and diminishing the cell proliferation capacity. However, patients receiving the R-CHOP treatment presented unfavorable prognoses in the presence of genotypes related to the intermediate production of IL-10 and high production of TGF-*β*1, indicating that cytokines may be related to response to treatment and action mechanisms of Rituximab. To confirm these findings, further studies with a larger number of samples are required. It should be noted that in our study, patients were well characterized and followed up during treatment. During the study period, all the individuals who presented the diagnosis of DLBCL and who agreed to participate in the study constituted our sample.

The results of this study indicate that polymorphisms in certain cytokine genes may favor the development of DLBCL and may indicate a better or worse prognosis in patients treated with R-CHOP.

## Figures and Tables

**Table 1:** Clinical and epidemiological characteristics of patients with diffuse large B-cell non-Hodgkin's lymphoma (*N* = 112) and controls (*N* = 221).

Characteristics	Patients	Controls	*P*
Age (mean ± standard deviation)	60.4 ± 15.4	50.6 ± 8.0	0.01
Gender *n* (%)			
Female	57 (50.9)	109 (49.3)	NS
Male	55 (49.1)	112 (50.7)	NS
Clinical stage *n* (%)			
I-II	44 (39.2)		
III-IV	68 (60.7)		
LDH *n* (%)			
≥Normal	51 (45.5)		
Unknown	8 (7.1)		
IPI *n* (%)			
Low and intermediate-low	70 (62.5)		
Intermediate-high and high	33 (29.5)		
Unknown	9 (8.0)		
Treatment *n* (%)			
R-CHOP	101 (90.2)		
Others	8 (7.1)		
Not treated	3 (2.7)		
Response to treatment with R-CHOP *n* (%)			
Complete remission	72 (71.2)		
Progression of disease	4 (4.0)		
Relapse	4 (4.0)		
Death	17 (16.8)		
Lost to follow-up	4 (4.0)		

NS = not significant; LDH = lactate dehydrogenase; IPI = international prognosis index; R-CHOP = Rituximab—Cyclophosphamide, Doxorubicin, Vincristine, and Prednisone.

**Table 2:** Distribution of the genotype frequencies in cytokine genes and the haplotype frequencies of *IL10* and *TGFB1* in patients with DLBCL and controls.

	Genotypes	Frequency		OR (95% CI)	*P*
		Patients *N* = 112 *n* (%)	Controls *N* = 221 *n* (%)		
Polymorphisms					
*TNF* -308G>A rs1800629	G/G	85 (75.9)	160 (72.4)		
	G/A	26 (23.2)	54 (24.4)		NS
	A/A	1 (0.9)	7 (3.2)		
*TGFB1* codon10T>C rs1982073	T/T	28 (25.0)	66 (29.9)		
	T/C	61 (54.5)	106 (48.0)		NS
	C/C	23 (20.5)	49 (22.2)		
*TGFB1* codon25G>C rs1800471	G/G	96 (85.7)	196 (88.7)		
	G/C	15 (13.4)	22 (9.9)		NS
	C/C	1 (0.9)	3 (1.4)		
*IL10* -1082A>G rs1800896	A/A	49 (43.8)	87 (39.4)		
	G/A	55 (49.1)	104 (47.1)		NS
	G/G	8 (7.1)	30 (13.6)		
*IL10* -819C>T rs1800871	C/C	36 (32.1)	101 (45.7)	1	
	C/T	58 (51.8)	90 (40.7)	2.23 (1.16-4.29)	0.013^∗^
	T/T	18 (16.1)	30 (13.6)		
*IL10* -592C>A rs1800872	C/C	6 (32.1)	101 (45.7)	1	
	C/A	58 (51.8)	90 (40.7)	2.23 (1.16-4.29)	0.013^∗^
	A/A	18 (16.1)	30 (13.6)		
*IL6* -174G>C rs1800795	G/G	59 (52.7)	95 (43.0)		
	G/C	41 (36.6)	99 (44.8)		NS
	C/C	12 (10.7)	27 (12.2)		
*IFNG* +874A>T rs2430561	A/A	57 (50.9)	61 (27.6)	1	
	A/T	38 (33.9)	117 (52.9)	0.35 (0.19-0.65)	<0.01^∗^
	T/T	17 (15.2)	43 (19.5)		

Haplotypes					
*IL10* -1082, -819, and -592	ATA	0.419	0.339	1	
	GCC	0.317	0.371		NS
	ACC	0.263	0.289	0.55 (0.32-0.95)	0.03
*TGFB1* codon10, codon25	TG	0.516	0.529	1	
	CG	0.409	0.407		NS
	CC	0.069	0.055		NS
	TC	0.006	0.009		NS

NS: not significant; *N*: population size; *n*: number of individuals with the allele or genotype; %: genotype and haplotype frequencies × 100; *P*: *P* value (adjusted by age and gender); OR: odds ratio; CI: confidence interval. ^∗^Best model: dominant—a single copy of the mutant allele is sufficient to modify the risk. The best inheritance model was chosen according to the lowest Akaike information criterion (AIC).

**Table 3:** Distribution of genotype frequencies in cytokine genes and in the haplotype frequencies of *IL10* and *TGFB1* in patients treated with R-CHOP and considering the prognosis of the disease.

	Genotypes	Frequency		OR (95% CI)	*P*
		Death, relapse, or progression	Complete remission		
		*N* = 25 *n* (%)	*N* = 72 *n* (%)		
Polymorphisms					
*TNF* -308G>A rs1800629	G/G	23 (92.0)	52 (72.2)		
	G/A	2 (8.0)	20 (27.8)		NS
	A/A				
*TGFB1* codon10T>C rs1982073	T/T-C/C	4 (16.0)	20 (27.8)	1.00	
	T/C	17 (68.0)	35 (48.6)	2.89 (1.04-8.05)	0.03^∗^
*TGFB1* codon25G>C rs1800471	G/G	19 (76.0)	64 (88.9)		
	G/C	5 (20.0)	8 (11.1)		NS
	C/C	1 (4.0)	0 (0)		
*IL10* -1082A>G rs1800896	A/A	13 (52.0)	28 (38.9)		
	G/A	12 (48.0)	37 (51.4)		NS
	G/G	0 (0)	7 (9.7)		
*IL10* -819C>T rs1800871	C/C	9 (36.0)	23 (31.9)		
	C/T	14 (56.0)	37 (51.4)		NS
	T/T	2 (8.0)	12 (16.7)		
*IL10* -592C>A rs1800872	C/C	9 (36.0)	23 (31.9)		
	C/A	14 (56.0)	37 (51.4)		NS
	A/A	2 (8.0)	12 (16.7)		
*IL6* -174G>C rs1800795	G/G	12 (48.0)	40 (55.6)		
	G/C	11 (44.0)	23 (31.9)		NS
	C/C	2 (8.0)	9 (12.5)		
*IFNG* +874A>T rs2430561	A/A	11 (44.0)	36 (50.0)		
	A/T	9 (36.0)	26 (36.1)		NS
	T/T	5 (20.0)	10 (13.9)		

Haplotypes					
*IL10* -1082, -819, and -592	ATA	0.360	0.424	1	
	GCC	0.240	0.354		NS
	ACC	0.400	0.222	2.83 (1.11-7.18)	0.03
*TGFB1* codon10, codon25	TG	0.510	0.500	1	
	CG	0.434	0.360		NS
	CC	0.045	0.140		NS
	TC	0.001	0		NS

NS: not significant; *N*: population size; *n*: number of individuals with the allele or genotype; %: genotype and haplotype frequencies × 100; *P*: *P* value (adjusted by age and gender); OR: odds ratio; CI: confidence interval. ^∗^Best model: overdominant—heterozygous alleles are compared to a pool of both homozygous alleles. The best inheritance model was chosen according to the lowest Akaike information criterion (AIC).

## Data Availability

The data used to support the findings of this are included within the article.

## Supplementary Materials

Supplementary Materials Supplementary Table 1: distribution of the genotype frequencies in cytokine genes in patients with DLBCL and controls (adjusted by age and sex).
