# Supplementary material for: Impact of SNPs/Haplotypes of IL10 and IFNG on the Development of Diffuse Large B-Cell Lymphoma
Source: J Immunol Res. 2019 Nov 26;2019:2137538. doi: 10.1155/2019/2137538 (PMC6899282; doi:10.1155/2019/2137538)
Supplement: Supplementary Materials — Supplementary Table 1: distribution of the genotype frequencies in cytokine genes in patients with DLBCL and controls (adjusted by age and sex). [file 2137538.f1.docx]

**Supplementary Material**

Supplementary Table 1. Distribution of the genotype frequencies in cytokine genes in patients with DLBCL and controls (adjusted by age and sex).

| **SNPs** | **Models** | **Genotypes** | **Patients N=112 n (%)** | **Controls N=221 n(%)** | **OR (95 CI)** | ***P*** | **AIC** |
| --- | --- | --- | --- | --- | --- | --- | --- |
| ***TNF*-308G>A rs1800629** | Codominant | G/G | 85 (75.9) | 160 (72.4) | 1 | 0.92 | 290.4 |
|  |  | G/A | 26 (23.2) | 54 (24.4) | 1.02 (0.51-2.05) |  |  |
|  |  | A/A | 1 (0.9) | 7 (3.2) | 0.66 (0.08-5.58) |  |  |
|  | Dominant | G/G | 85 (75.9) | 160 (72.4) | 1 | 0.96 | 288.6 |
|  |  | G/A-A/A | 27 (24.1) | 61 (27.6) | 0.98 (0.50-1.93) |  |  |
|  | Recessive | G/G-G/A | 111 (99.1) | 214 (96.8) | 1 | 0.69 | 288.4 |
|  |  | A/A | 1 (0.9) | 7 (3.2) | 0.66 (0.08-5.51) |  |  |
|  | Overdominant | G/G-A/A | 86 (76.8) | 167 (75.6) | 1 | 0.93 | 288.6 |
|  |  | G/A | 26 (23.2) | 54 (24.4) | 1.03 (0.51-2.07) |  |  |
|  | Log-additive |  |  |  | 0.95 (0.53-1.72) | 0.86 | 288.5 |
| ***TGFB1*codon10T>C rs1982073** | Codominant | T/T | 28 (25) | 66 (29.9) | 1 | 0.26 | 287.9 |
|  |  | T/C | 61 (54.5) | 106 (48) | 1.29 (0.64-2.62) |  |  |
|  |  | C/C | 23 (20.5) | 49 (22.2) | 0.66 (0.25-1.68) |  |  |
|  | Dominant | T/T | 28 (25) | 66 (29.9) | 1 | 0.81 | 288.5 |
|  |  | T/C-C/C | 84 (75) | 155 (70.1) | 1.09 (0.55-2.13) |  |  |
|  | Recessive | T/T-T/C | 89 (79.5) | 172 (77.8) | 1 | 0.14 | 286.4 |
|  |  | C/C | 23 (20.5) | 49 (22.2) | 0.55 (0.24-1.25) |  |  |
|  | Overdominant | T/T-C/C | 51 (45.5) | 115 (52) | 1 | 0.17 | 286.7 |
|  |  | T/C | 61 (54.5) | 106 (48) | 1.53 (0.83-2.82) |  |  |
|  | Log-additive |  |  |  | 0.86 (0.56-1.33) | 0.49 | 288.1 |
| ***TGFB1*codon25G>C rs1800471** | Codominant | G/G | 96 (85.7) | 196 (88.7) | 1 | 0.7 | 289.9 |
|  |  | G/C | 15 (13.4) | 22 (9.9) | 1.25 (0.49-3.19) |  |  |
|  |  | C/C | 1 (0.9) | 3 (1.4) | 0.33 (0.01-9.81) |  |  |
|  | Dominant | G/G | 96 (85.7) | 196 (88.7) | 1 | 0.79 | 288.5 |
|  |  | G/C-C/C | 16 (14.3) | 25 (11.3) | 1.13 (0.46-2.80) |  |  |
|  | Recessive | G/G-G/C | 111 (99.1) | 218 (98.6) | 1 | 0.48 | 288.1 |
|  |  | C/C | 1 (0.9) | 3 (1.4) | 0.32 (0.01-9.54) |  |  |
|  | Overdominant | G/G-C/C | 97 (86.6) | 199 (90) | 1 | 0.62 | 288.3 |
|  |  | G/C | 15 (13.4) | 22 (9.9) | 1.27 (0.50-3.22) |  |  |
|  | Log-additive |  |  |  | 1.02 (0.45-2.28) | 0.97 | 288.6 |
| ***IL10*-1082A>G rs1800896** | Codominant | A/A | 49 (43.8) | 87 (39.4) | 1 | 0.44 | 288.9 |
|  |  | G/A | 55 (49.1) | 104 (47.1) | 0.71 (0.38-1.35) |  |  |
|  |  | G/G | 8 (7.1) | 30 (13.6) | 0.58 (0.20-1.66) |  |  |
|  | Dominant | A/A | 49 (43.8) | 87 (39.4) | 1 | 0.22 | 287.1 |
|  |  | G/A-G/G | 63 (56.2) | 134 (60.6) | 0.69 (0.37-1.26) |  |  |
|  | Recessive | A/A-G/A | 104 (92.9) | 191 (86.4) | 1 | 0.45 | 288 |
|  |  | G/G | 8 (7.1) | 30 (13.6) | 0.69 (0.25-1.87) |  |  |
|  | Overdominant | A/A-G/G | 57 (50.9) | 117 (52.9) | 1 | 0.47 | 288 |
|  |  | G/A | 55 (49.1) | 104 (47.1) | 0.80 (0.43-1.47) |  |  |
|  | Log-additive |  |  |  | 0.74 (0.47-1.18) | 0.21 | 287 |
| ***IL10*-819C>T rs1800871** | Codominant | C/C | 36 (32.1) | 101 (45.7) | 1 | **0.03** | 283.9 |
|  |  | C/T | 58 (51.8) | 90 (40.7) | **2.06 (1.03-4.11)** |  |  |
|  |  | T/T | 18 (16.1) | 30 (13.6) | 2.80 (1.16-6.78) |  |  |
|  | Dominant | C/C | 36 (32.1) | 101 (45.7) | 1 | **0.013** | 282.4 |
|  |  | C/T-T/T | 76 (67.9) | 120 (54.3) | **2.23 (1.16-4.29)** |  |  |
|  | Recessive | C/C-C/T | 94 (83.9) | 191 (86.4) | 1 | 0.12 | 286.2 |
|  |  | T/T | 18 (16.1) | 30 (13.6) | 1.88 (0.86-4.07) |  |  |
|  | Overdominant | C/C-T/T | 54 (48.2) | 131 (59.3) | 1 | 0.2 | 286.9 |
|  |  | C/T | 58 (51.8) | 90 (40.7) | 1.48 (0.81-2.71) |  |  |
|  | Log-additive |  |  |  | **1.72 (1.12-2.63)** | **0.014** | 282.5 |
| ***IL10*-592C>A rs1800872** | Codominant | C/C | 36 (32.1) | 101 (45.7) | 1 | **0.03** | 283.9 |
|  |  | C/A | 58 (51.8) | 90 (40.7) | **2.06 (1.03-4.11)** |  |  |
|  |  | A/A | 18 (16.1) | 30 (13.6) | 2.80 (1.16-6.78) |  |  |
|  | Dominant | C/C | 36 (32.1) | 101 (45.7) | 1 | **0.013** | 282.2 |
|  |  | C/A-A/A | 76 (67.9) | 120 (54.3) | **2.23 (1.16-4.29)** |  |  |
|  | Recessive | C/C-C/A | 94 (83.9) | 191 (86.4) | 1 | 0.12 | 286.2 |
|  |  | A/A | 18 (16.1) | 30 (13.6) | 1.88 (0.86-4.07) |  |  |
|  | Overdominant | C/C-A/A | 54 (48.2) | 131 (59.3) | 1 | 0.2 | 286.9 |
|  |  | C/A | 58 (51.8) | 90 (40.7) | 1.48 (0.81-2.71) |  |  |
|  | Log-additive |  |  |  | **1.72 (1.12-2.63)** | **0.014** | 282.3 |
| ***IL6*174G>C rs1800795** | Codominant | G/G | 59 (52.7) | 95 (43) | 1 | 0.16 | 286.9 |
|  |  | G/C | 41 (36.6) | 99 (44.8) | 0.53 (0.28-1.03) |  |  |
|  |  | C/C | 12 (10.7) | 27 (12.2) | 0.64 (0.24-1.74) |  |  |
|  | Dominant | G/G | 59 (52.7) | 95 (43) | 1 | 0.058 | 285 |
|  |  | G/C-C/C | 53 (47.3) | 126 (57) | 0.56 (0.30-1.02) |  |  |
|  | Recessive | G/G-G/C | 100 (89.3) | 194 (87.8) | 1 | 0.73 | 288.5 |
|  |  | C/C | 12 (10.7) | 27 (12.2) | 0.85 (0.32-2.20) |  |  |
|  | Overdominant | G/G-C/C | 71 (63.4) | 122 (55.2) | 1 | 0.088 | 285.7 |
|  |  | G/C | 41 (36.6) | 99 (44.8) | 0.58 (0.31-1.10) |  |  |
|  | Log-additive |  |  |  | 0.69 (0.44-1.10) | 0.12 | 286.1 |
| ***IFNG*+874A>T rs2430561** | Codominant | A/A | 57 (50.9) | 61 (27.6) | 1 | **0.0023** | 278.4 |
|  |  | T/A | 38 (33.9) | 117 (52.9) | **0.30 (0.15-0.60)** |  |  |
|  |  | T/T | 17 (15.2) | 43 (19.5) | 0.47 (0.20-1.10) |  |  |
|  | Dominant | A/A | 57 (50.9) | 61 (27.6) | 1 | **0.0008** | 277.4 |
|  |  | T/A-T/T | 55 (49.1) | 160 (72.4) | **0.35 (0.19-0.65)** |  |  |
|  | Recessive | A/A-T/A | 95 (84.8) | 178 (80.5) | 1 | 0.73 | 288.5 |
|  |  | T/T | 17 (15.2) | 43 (19.5) | 0.87 (0.40-1.92) |  |  |
|  | Overdominant | A/A-T/T | 74 (66.1) | 104 (47.1) | 1 | **0.0027** | 279.6 |
|  |  | T/A | 38 (33.9) | 117 (52.9) | **0.39 (0.20-0.73)** |  |  |
|  | Log-additive |  |  |  | **0.57 (0.37-0.90)** | **0.013** | 282.4 |

N: population size; n: number of individuals with the allele; %: genotype frequencies x100; *P*: *P*-value; OR: odds ratio; CI: confidence interval; AIC: Akaike information criterion value.
